# Huaier extract restrains the proliferative potential of endocrine-resistant breast cancer cells through increased ATM by suppressing miR-203

**DOI:** 10.1038/s41598-017-07550-9

**Published:** 2017-08-04

**Authors:** Sumei Gao, Xiaoyan Li, Xia Ding, Liyu Jiang, Qifeng Yang

**Affiliations:** 1 Department of Breast Surgery, Qilu Hospital, Shandong University, Jinan, P.R. China; 2 Department of Oncology, Qilu Hospital, Shandong University, Jinan, P.R. China; 3 Pathology Tissue Bank, Qilu Hospital, Shandong University, Jinan, P.R. China

## Abstract

Endocrine therapy is one of the main treatments for breast cancer patients in the early stages. Tamoxifen and fulvestrant are the major drugs of endocrine therapy for breast cancer patients. However, acquired drug resistance often caused treatment failure and relapse for patients, which is a major clinical problem. We investigated whether Huaier extract had effects on endocrine-resistant breast cancer cells. In our study, we aimed to demonstrate the inhibitory effects of Huaier extract on tamoxifen-resistant cells (M7-TR) and fulvestrant-resistant cells (M7-FR). Using MTT and clone formation assays, we found that Huaier extract could inhibit the proliferation in M7-TR and M7-FR cells. Flow cytometry and western blotting illustrated that Huaier extract could induce G0/G1 arrest in both endocrine-resistant breast cancer cells. Mechanistically, we present that Huaier extract significantly increased ataxia telangiectasia mutation (ATM) via down-regulation of miR-203. Huaier extract also had the inhibitory effects on tumour growth *in vivo* in a xenograft mouse model. These results demonstrated that Huaier extract could inhibit the proliferation of M7-TR and M7-FR cells by increasing ATM via suppression of miR-203.

## Introduction

Breast cancer has become the most common malignancy in women in a variety of countries. In 2015, approximately 4,292,000 Chinese women were diagnosed with breast cancer, and 2,814,000 died from this disease^1^. Approximately 75% of women with breast cancer are hormone receptor positive, expressing estrogen receptor (ER), and endocrine therapies are effective for patients with ER-positive breast cancer^2^. The selective estrogen receptor modulator (SERM) tamoxifen (TAM)^3^ and selective estrogen receptor down-regulator (SERD) fulvestrant^4^ are the major endocrine therapy drugs for breast cancer patients in early stages. Unfortunately, more than 50% of patients with initially responding tumours eventually experience tumour relapse and die from acquired resistance^5^. Therefore, there is a great need for new agents and therapeutic regimens for acquired endocrine-resistance patients.

Traditional Chinese medicine (TCM) has a long history of three millennia in Asia^6^. Huaier extract is one of the typical TCMs, and it has shown anti-tumour effects in several cancers^7^. In our previous study, several mechanisms of the anti-tumour effects of Huaier extract were demonstrated, such as anti-angiogenesis, reversal of drug-resistance, radiosensitization, anti-metastasis and activation of the immune system^6, 8–11^. In recent years, Huaier extract has been routinely used as a complementary drug for breast cancer.

Several mechanisms contribute to endocrine resistance, including apoptosis^12^, autophagy^13^, and DNA damage^14^. Ataxia telangiectasia mutation (ATM) belongs to a family of phosphatidylinositol 3-kinases that regulate cell cycle checkpoints and DNA recombination and repair^15^. ATM is a key regulator of the DNA damage response pathway^16^. ATM was negatively correlated with tumourigenesis and invasiveness^17^. A previously study demonstrated that ATM was a direct target of miR-203 in colorectal cancer cells^18^. In this study, we found that Huaier extract demonstrated proliferation-inhibiting effects related to the cell cycle and DNA damage. We further investigated the roles of miR-203 and ATM in endocrine-resistant breast cancer cells after Huaier extract treatment.

## Results

### Huaier extract inhibited proliferation of endocrine-resistant breast cancer cells

**Figure 1:**
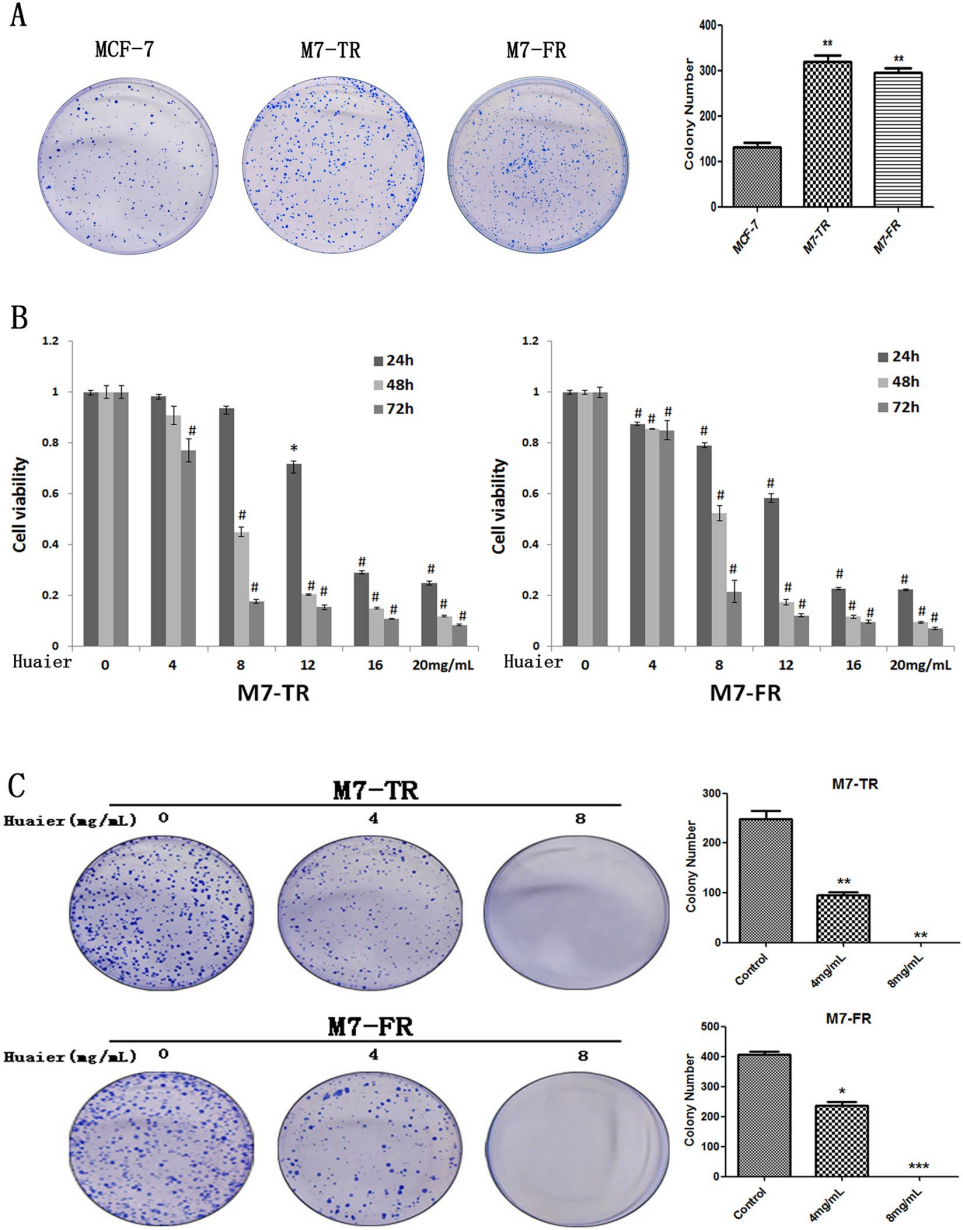
The effects of the indicated concentrations of Huaier extract on cell viability and clone formation capacity in M7-TR and M7-FR cells. (**A**) Cell proliferation of endocrine-resistant breast cancer cells (M7-TR and M7-FR) and endocrine-sensitive breast cancer cells (MCF-7). (**B**) The effects of Huaier extract on cell viability were measured by MTT assay. Huaier extract inhibited cell viability in both cell lines. (**C**) Representative images and the colony formation rates of M7-TR and M7-FR cell colonies after treatment with Huaier extract for 24 h. *P < 0.05 or ^#^ *P* < 0.01, compared with the controls. The data are presented as the mean ± SD of three separate experiments.

Clone formation assay was performed to verify the proliferative ability between endocrine-resistant breast cancer cells (M7-TR and M7-FR) and endocrine-sensitive breast cancer cells (MCF-7). As shown in Fig. 1A, the growth of MCF-7 cells was significantly slower than that of M7-TR and M7-FR cells. The anti-proliferative effects of Huaier extract on both resistant breast cancer cells were investigated by MTT assay and clone formation assays. As shown in Fig. 1B, Huaier extract inhibited the cell viability of both M7-TR and M7-FR cells. In both the M7-TR and M7-FR cells, a sharp decrease in cell viability was present at 8 mg/mL, independent of the treatment time. The M7-TR and M7-FR cells indicated cytotoxic effects more evidently at 48 and 72 h. As shown in Fig. 1C, a significant inhibitory effect was observed with different concentrations of Huaier extract. Huaier extract could significantly inhibit clone formation in both M7-TR and M7-FR cells.

### Huaier extract induced G0/G1 cell cycle arrest in endocrine-resistant breast cancer cells

**Figure 2:**
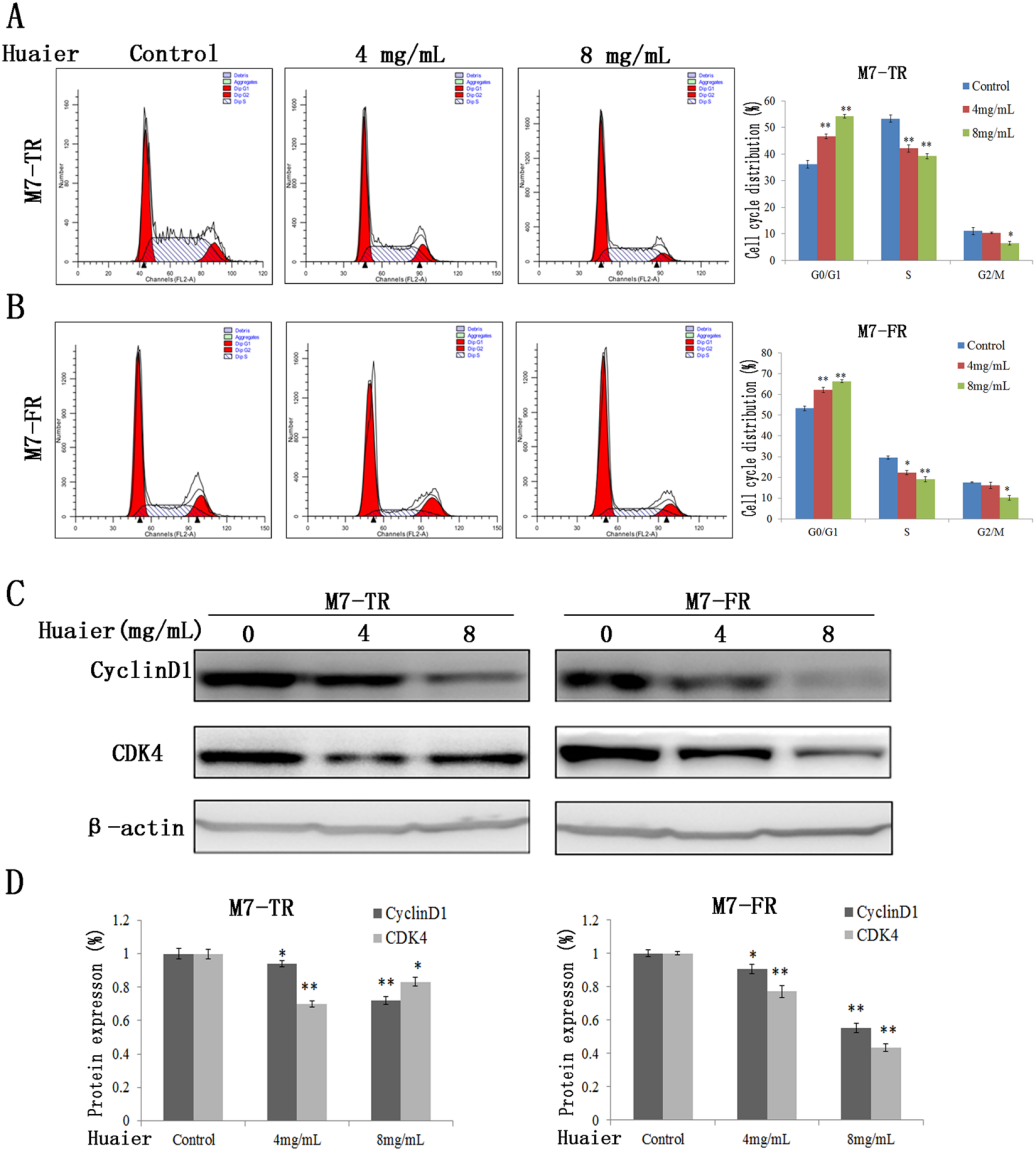
Induction of G0/G1 phase arrest in M7-TR and M7-FR cell lines by Huaier extract. The DNA content of M7-TR (**A**) and M7-FR (**B**) cells, treated as above for 48 h, was detected and analysed by flow cytometry. The data represent the results of three independent experiments; *P < 0.05 or **P < 0.01. Changes in cell cycle progression could be due to cyclin D1 and CDK4 in the M7-TR (**E**) and M7-FR (**F**) cells. The data are presented as the mean ± SD of three separate experiments.

The effects of Huaier extract on cell cycle progression in M7-TR and M7-FR cells were investigated by flow cytometry. Cells were exposed to different concentrations (0, 4 or 8 mg/mL) of Huaier extract for 24 h. After examination with a flow cytometer, the results demonstrated that Huaier extract resulted in a significant accumulation of cells in the G0/G1 phase. In M7-TR cells, the percentage of cells in the G0/G1 phase increased from 36.26 to 54.3% upon Huaier extract treatment (Fig. 2A). As shown in Fig. 2B, cells treated with Huaier extract had increasing percentages of cells in the G0/G1 phase (from 53.29 to 66.39%). To investigate the mechanism of G0/G1 arrest, we detected the protein levels of cell cycle-regulating proteins by western blotting. We detected two important cell cycle-regulating proteins, cyclin D1 and CDK4, which were obviously decreased in both cell lines following treatment with Huaier extract, which might explain the G0/G1 cell cycle arrest for both cell lines (Fig. 2C and D).

### Huaier extract up-regulated ATM in endocrine-resistant breast cancer cells

**Figure 3:**
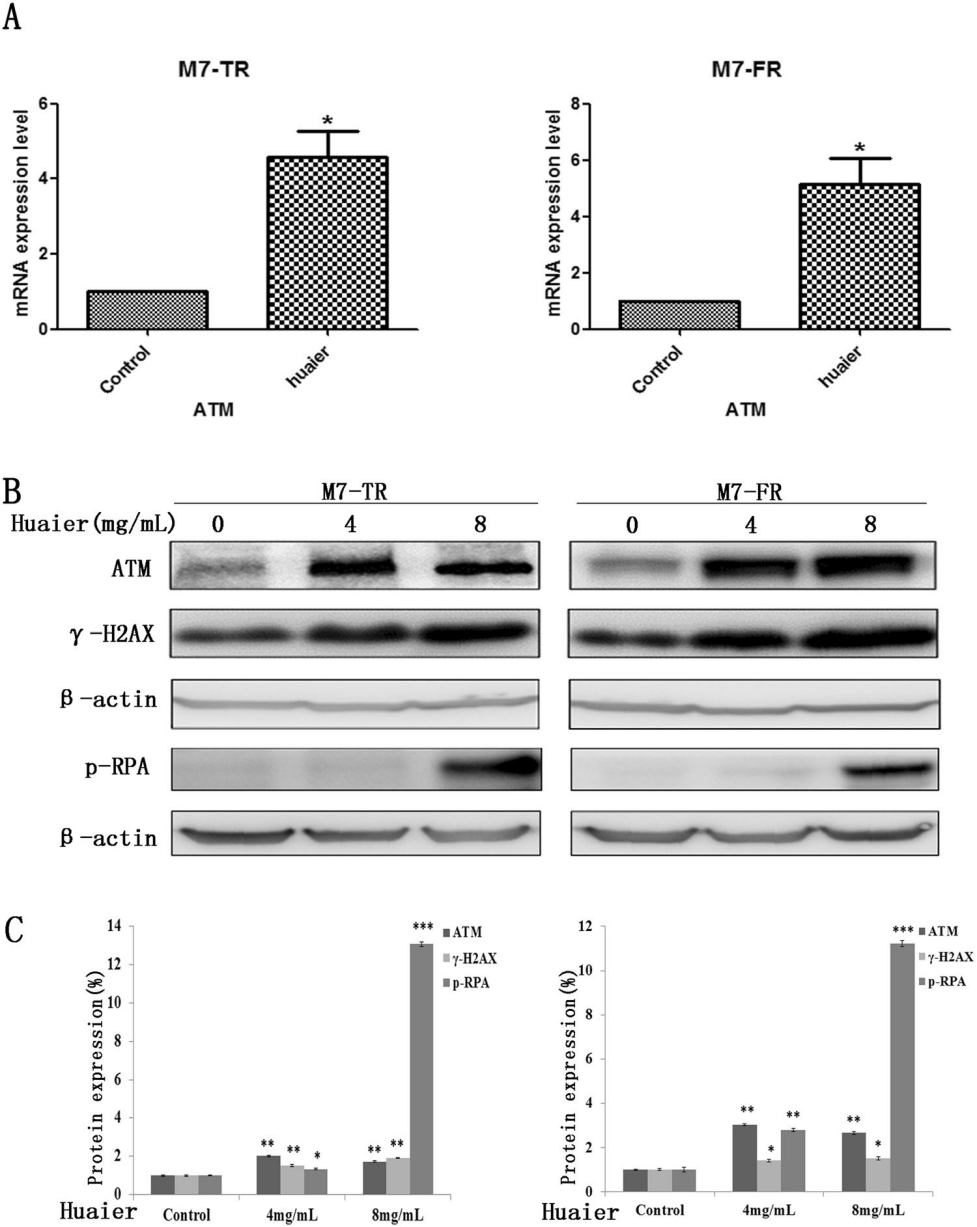
Changes in ATM, γ-H2AX and p-RPA in M7-TR and M7-FR cells after treatment with Huaier extract for 48 h. (**A**) M7-TR and M7-FR cells were treated with Huaier extract (8 mg/mL) for 48 h. The mRNA levels were detected by qRT-PCR. (**B** and **C**) Changes in protein levels in M7-TR and M7-FR cells after treatment with different concentration of Huaier extract for 48 h. *P < 0.05 or **P < 0.01, compared with the controls. The data are presented as the mean ± SD of three separate experiments.

To clarify the molecular mechanism of the inhibition of proliferation of M7-TR and M7-FR cells, we detected ATM in M7-TR and M7-FR using qRT-PCR and western blot assays. Cells were incubated with Huaier extract (8 mg/mL) for 48 h. Compared to the controls, Huaier extract could significantly increase the mRNA levels of ATM in M7-TR and M7-FR cells (Fig. 3A). γ-H2AX was phosphorylated by ataxia-telangiectasia mutation (ATM)^19^. Replication protein A (RPA), the major cellular single-stranded DNA (ssDNA)–binding protein complex, was downstream target of ATM^20^. As shown in Fig. 3B and C, the protein levels of ATM and p-RPA increased in both M7-TR and M7-FR cells by treatment with different concentrations of Huaier extract (4 or 8 mg/mL). γ-H2AX was increased significantly in M7-TR cells.

### ATM was regulated by miR-203 in endocrine-resistant breast cancer cells

**Figure 4:**
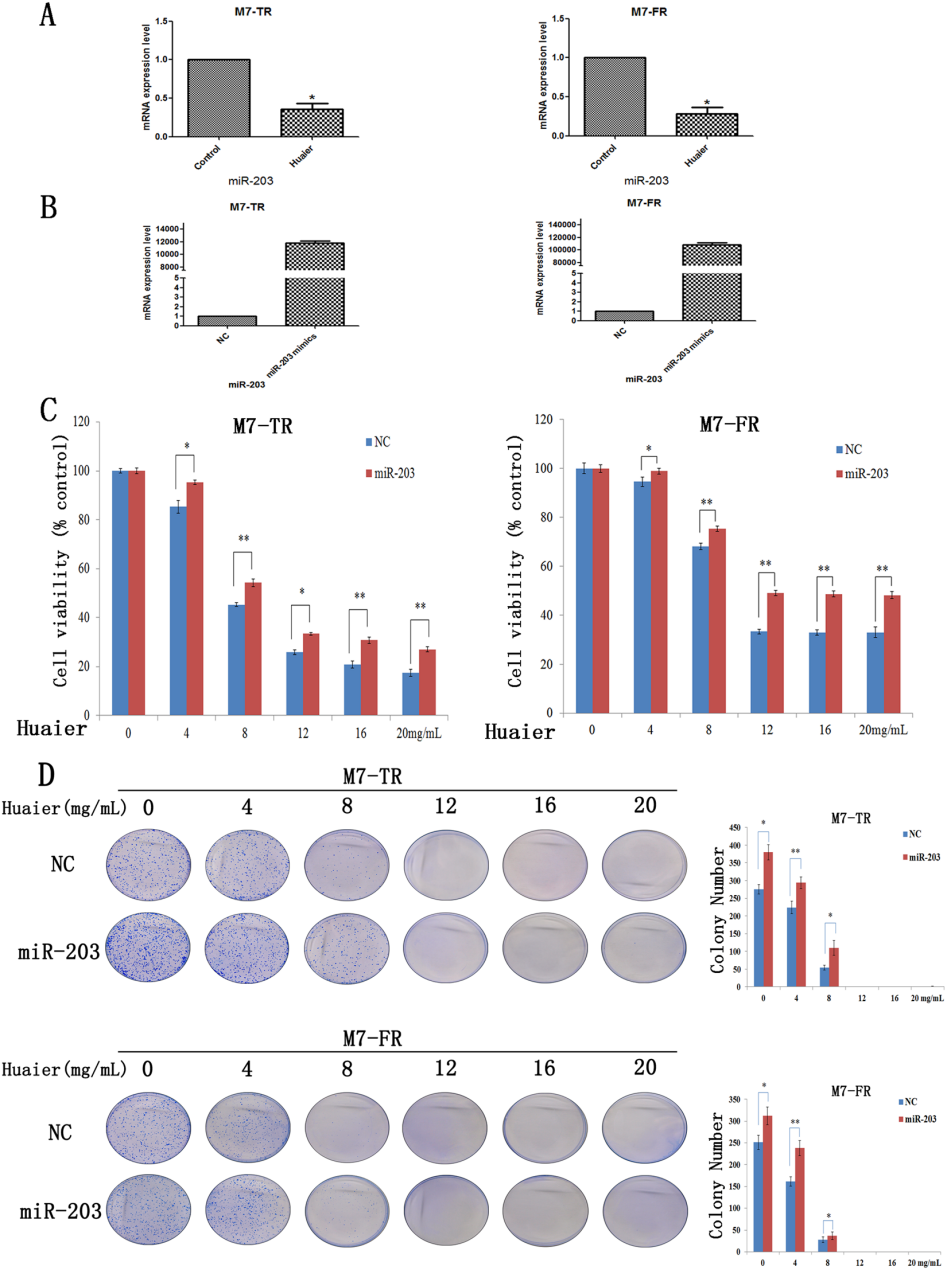
miR-203 is downregulated by Huaier extract in M7-TR and M7-FR cells. (**A**) Several miRNAs were detected in M7-TR and M7-FR after treatment with Huaier extract for 48 h. (**B**) miR-230 overexpressed by miR-203 mimics in M7-TR and M7-FR cells. (**C**) Overexpression of miR-203 in M7-TR and M7-FR cells; MTT assay was performed to detect the cell viability after treatment with different concentrations of Huaier extract for 48 h. (**D**) Overexpression of miR-203 in M7-TR and M7-FR cells. Representative images and the colony formation rate of M7-TR and M7-FR cell colonies after treatment with Huaier extract for 24 h. *P < 0.05 or **P < 0.01, compared with the controls. The data are presented as the mean ± SD of three separate experiments.

To explore the regulation of ATM, we detected miR-203 expression based on data mining and miRNA prediction. We used the bioinformatics algorithms Targetscan, miRbase and PicTar to search for the regulator of ATM. As shown in Fig. 4A, Huaier extract significantly decreased miR-203 in both M7-TR and M7-FR cells. We further analysed whether Huaier extract inhibited the proliferation of M7-TR and M7-FR cells through miR-203. In M7-TR and M7-FR cells, miR-203 was over-expressed and confirmed the efficacy of transfection by qRT-PCR (Fig. 4B). After transfection for 48 h, MTT and clone formation assays were used to measure cell viability. As shown in Fig. 4C, overexpression of miR-203 led to decreased anti-proliferative activity of Huaier extract. Clone formation assay revealed that overexpression of miR-203 increased the clone formation ability (4 mg/mL and 8 mg/mL) of both cancer cell lines (Fig. 4D).

### Huaier extract increased ATM by suppressing miR-203 in endocrine-resistant breast cancer cells

**Figure 5:**
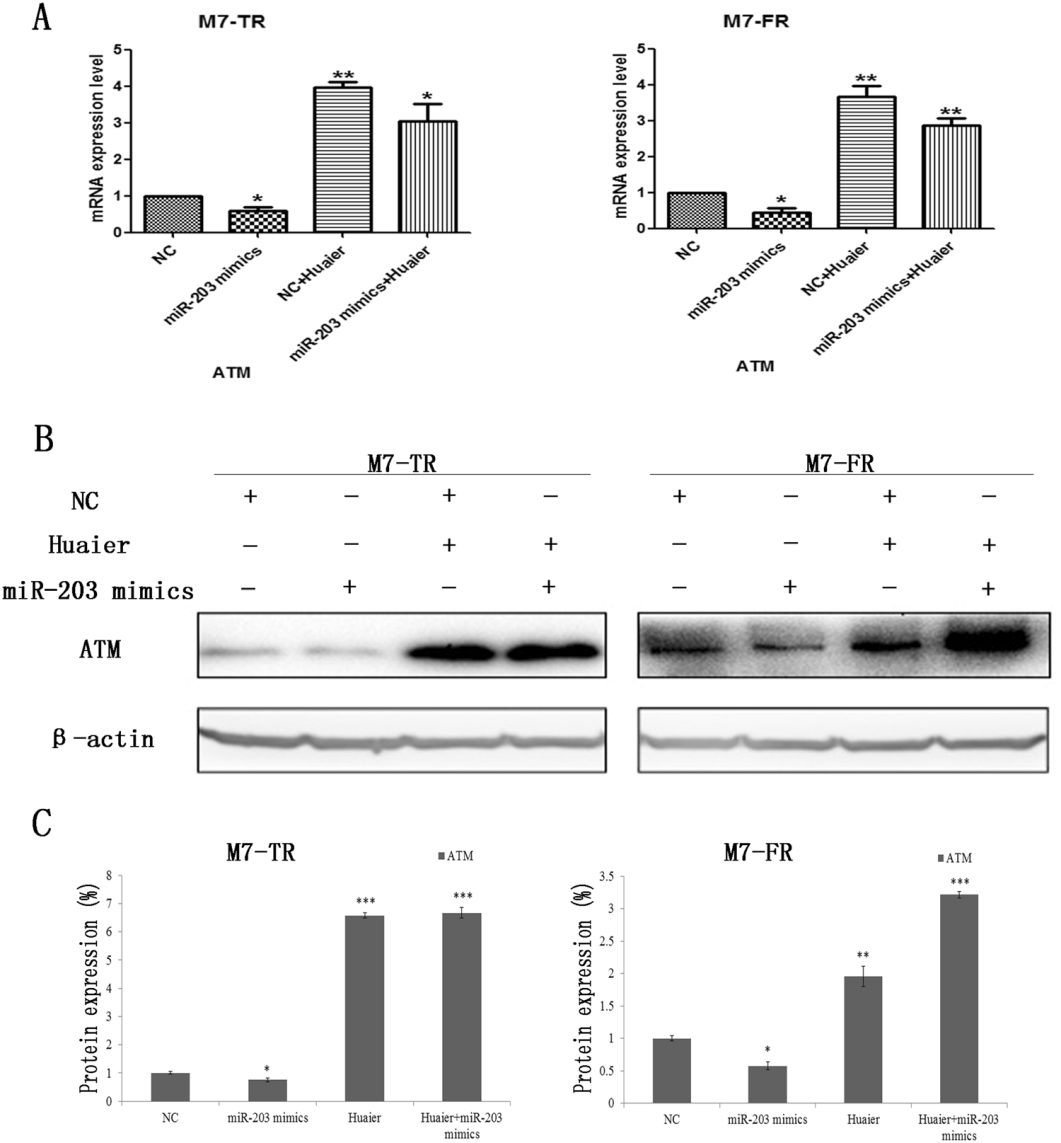
Huaier extract up-regulated ATM by suppressing miR-203 in M7-TR and M7-FR cells. (**A**) qRT-PCR analysis demonstrated that the transfection of miR-203 mimics reversed the mRNA changes of ATM induced by combined treatment with Huaier extract. (**B** and **C**) The protein expression levels of ATM after transfection of miR-203 mimics alone or combined with Huaier extract in M7-TR and M7-FR cells. *P < 0.05 or **P < 0.01, compared with the controls. The data are presented as the mean ± SD of three separate experiments.

To explore the further mechanism between ATM and miR-203 in M7-TR and M7-FR cells, we transfected miR-203 mimics to over-express miR-203 in both cell lines. Subsequently, cells were treated with Huaier extract or not for 48 h. As shown in Fig. 5A, the mRNA level of ATM was detected by qRT-PCR. Overexpression of miR-203 significantly reversed the expression of ATM in both cell lines. Compared to controls, ATM was increased after being combined with Huaier extract. To further confirm this finding, western blot analysis confirmed that miR-203 overexpression increased the level of ATM after combination with Huaier extract (Fig. 5B and C).

### Huaier extract inhibited the growth of subcutaneous tumours

**Figure 6:**
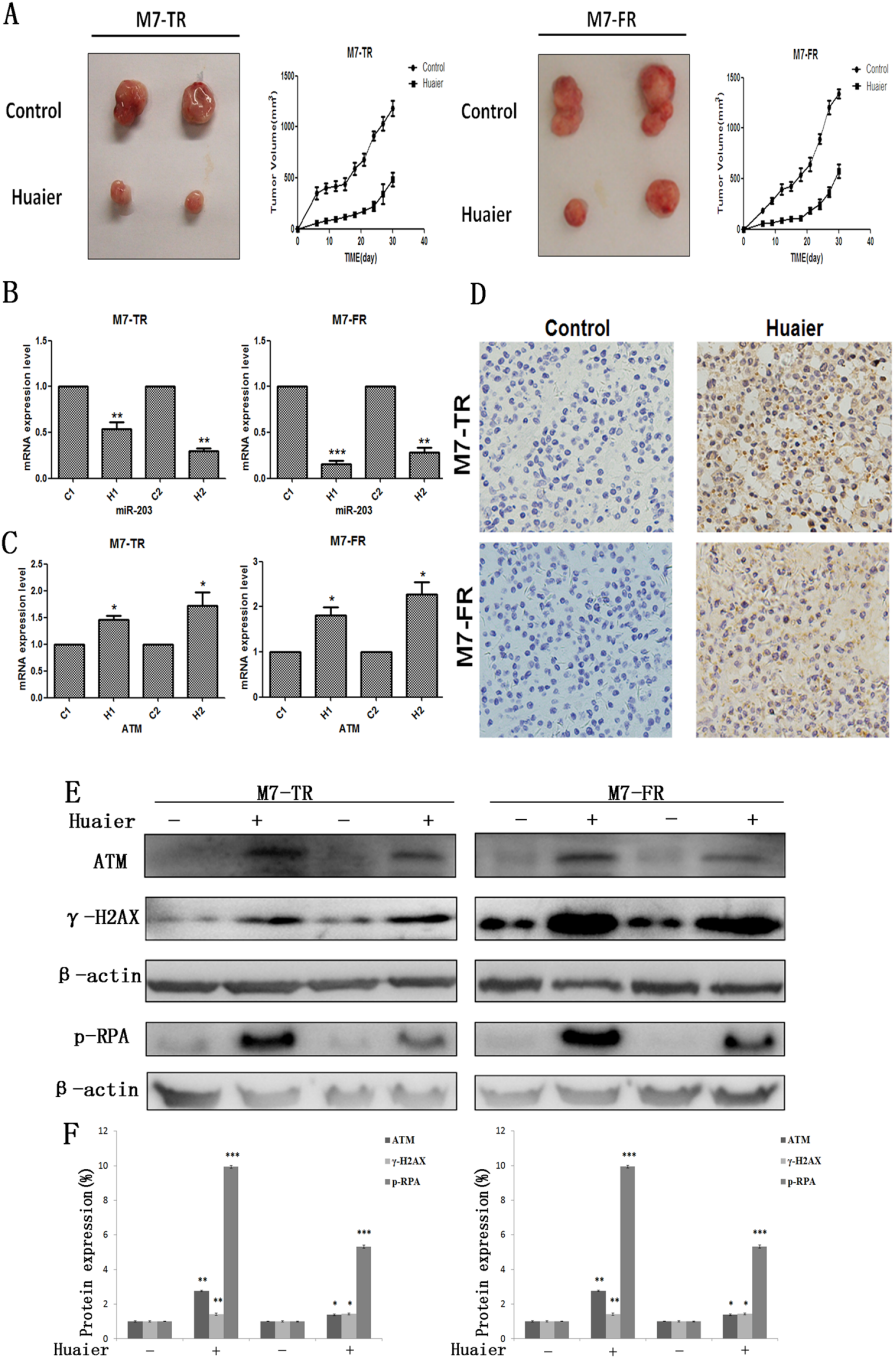
Huaier extract enhanced the suppression of tumourigenesis in a xenograft model. (**A**) Flank tumours were established in BALB/c nu/nu female mice, as described in the Materials and Methods section. The mice were sacrificed 40 days after flank injection; representative tumours are shown. Growth curves of xenograft tumours after injection of M7-TR and M7-FR cells. The miR-203 (**B**) and ATM (**C**) expression levels after treatment or not with Huaier extract. (**D**) Immunohistochemical analysis of ATM. (**E** and **F**) The protein changes of ATM, γ-H2AX and p-RPA after treatment with Huaier extract, compared to the control group. *P < 0.05 or **P < 0.01 compared with the controls. The data are presented as the mean ± SD of three separate experiments.

To demine the curative effect of Huaier extract *in vivo*, M7-TR and M7-FR cells were subcutaneously injected into the right flanks of BALB/c nu/nu mice. As shown in Fig. 6A, xenograft tumour growth was reduced after Huaier extract treatment, compared to in the control group. To further investigate the mechanism underlying the inhibition of tumour growth by Huaier extract *in vivo*, we detected the miR-203 (Fig. 6B) and ATM (Fig. 6C) mRNA levels using qRT-PCR. Furthermore, we measured ATM and γ-H2AX protein levels using immunohistochemical staining and western blot analysis. Huaier extract increased ATM expression levels (Fig. 6D). Compared to controls, ATM, γ-H2AX and p-RPA were increased by treatment with Huaier extract (Fig. 6E and F). These results demonstrated that Huaier extract had inhibitory effects on endocrine resistance *in vivo*.

## Discussion

Endocrine therapy has been the first-line treatment for hormone-positive breast cancer patients over the past 30 years^21, 22^. However, acquired endocrine resistance is a serious problem in clinical therapy. Acquired endocrine resistance often causes failure of endocrine therapy and relapse during clinical therapy^22^. Increasing research has investigated the underlying mechanism of endocrine resistance to search for a new therapeutic target or biomarker, including regulators of the ER pathway^23^ and modulations in the cell cycle and apoptotic machinery^24^. In this study, we revealed that Huaier extract, which has been widely used for antitumour effects in several cancers, inhibited proliferation in endocrine-resistant breast cancer cells.

Accumulating evidence has underscored that TCM is a rich source for finding new drugs, and increasingly, TCM has been found to have anti-tumour effects on cancer^25, 26^. Furthermore, TCM has revealed various effects on drug or radiation resistance^27–29^. Huaier extract is classified as an official fungus, and it has been widely used as a complementary agent for cancer therapy in recent years^30^. In a previous study, our group revealed that Huaier extract showed strong anti-proliferation effects on breast cancer^31^ and synergized with endocrine drugs and radiosensitization via various pathways^10, 32^. As shown in MTT and clone formation assays, we found that Huaier extract had strong inhibitory effects of the proliferation of M7-TR and M7-FR cells. Our founding revealed that Huaier extract induced G0/G1 cell cycle arrest. Cell cycle arrest is often related to cell cycle-regulating proteins. Cyclin D1 is an important checkpoint in cell cycle progression in G1 to S transition by binding to CDK4, which is required for transition from the G1 to S phase^33^. In this study, Cyclin D1 and CDK4 were decreased by treatment with Huaier extract.

Currently, DNA damage was negatively regulated for tumourigenesis and drug resistance in cancer^34, 35^. Ataxia telangiectasia mutation (ATM) is a tumour-suppressor gene encoding a serine/threonine kinase, and it plays a key role in DNA double-strand breaks (DSBs) and activation of cell cycle checkpoints^17, 36^. As measured by western blot, Huaier extract could significantly increase ATM and p-RPA in M7-TR and M7-FR cells. In previous studies, ATM was regulated by several miRNAs, such as miR-203, in various cancers^18, 37^. Our study demonstrated that Huaier extract up-regulated ATM by inhibiting miR-203 in M7-TR cells. Overexpression of miR-203 could not significantly inhibit ATM expression in M7-FR cells (Fig. 5B). Therefore, there are likely other functional mediators of Huaier extract’s effects on M7-FR cells. In addition, xenograft tumourigenicity assay demonstrated that Huaier extract could inhibit the tumourigenesis of endocrine-resistant cells *in vivo*.

In conclusion, our results demonstrated that Huaier extract has effects on cell cycle arrest by regulating cell cycle-related proteins, and it induced DNA damage through the miR-203/ATM pathway *in vitro* and *in vivo*. These data suggested that Huaier extract should be investigated as a potential supplementary drug for breast cancer patients with endocrine resistance. Additionally, the effects of Huaier extract on endocrine-resistant breast cancer cells in other molecular mechanisms, such as apoptosis, autophagy, tumour-associated macrophages, should be investigated in further studies. However, further clinical correlation studies should also assess the toxicity and efficacy of Huaier extract treatment, and our current study could provide the proof suggesting that Huaier extract is promising for further clinical investigation in breast cancer patients with endocrine resistance.

## Materials and Methods

### Cell lines and reagents

Tamoxifen-resistant cells (M7-TR) were developed by culturing tamoxifen-sensitive MCF-7 cells in the presence of 10 μM tamoxifen for more than 1 year. Fulvestrant-resistant cells (M7-FR) were developed by culturing fulvestrant-sensitive MCF-7 cells in the presence of 175 nM fulvestrant for more than 1 year. The antibodies utilized included anti-Cyclin D1, CDK4 and ATM, obtained from Cell Signaling Technology (Beverly, MA, USA). Mouse anti-γ-H2AX antibody (Millipore, Billerica, MA, USA), rabbit anti-p-RPA antibody (Abcam), tamoxifen, fulvestrant and mouse monoclonal antibody against β-actin were purchased from Sigma-Aldrich (St. Louis, MO, USA). The streptavidin–peroxidase–biotin reagent kit was acquired from Zhongshan Biotechnology (Beijing, China). Huaier aqueous extract, a kind gift of Gaitianli Medicine Co. Ltd. (Jiangsu, China), was dissolved in DMEM in a storage concentration of 100 mg/mL. All of the other chemicals were obtained from Sigma-Aldrich (St. Louis, MO, USA) unless specifically described.

### Cell culture

M7-TR and M7-FR cells were routinely cultured in Dulbecco’s modified Eagle’s medium (DMEM, Gibco, Rockville, IN, USA) containing 10% foetal bovine serum (FBS, Clark Bioscience, Seabrook, MD, USA). Penicillin at a dose of 100 U/ml and 100 µg/ml streptomycin were used in 5% CO_2_ at 37 °C. The medium for M7-TR cell lines was supplemented with 10 μM tamoxifen, and for M7-FR cell lines, it was supplemented with 175 nM fulvestrant.

### Cell proliferation assay

Colony formation assay was used to assess cell proliferation. The cells were seeded at a density of 1.5 × 10^3^ cells in a 6-cm Petri dish. The cells were cultured for another 14 days. The medium was refreshed every three days. Thereafter, the cells were washed with phosphate-buffered saline (PBS) and were fixed with paraformaldehyde and stained with 0.5% crystal violet. Images of clones were obtained using an Olympus digital camera (Olympus, Tokyo, Japan).

### Cell viability assay

Cell viability was examined by 3-(4,5-dimethylthiazol-2-yl)-2,5-diphenyltetrazolium bromide (MTT) assay. M7-TR and M7-FR (3 × 10^3^ cells/well) cells were seeded into 96-well culture plates and were incubated in 5% CO_2_ at 37 °C. After incubation overnight, the cells were exposed to vehicle or Huaier extract and were incubated for 24, 48, and 72 h. Then, 20 μL of MTT (5 mg/mL) were added to each well, and the cells were incubated for another 4 h at 37 °C. After removal of MTT, 100 μL of DMSO were added to each well, and the plate was gently shaken for 10 min at room temperature. The absorbance was measured by a Microplate Reader (Bio-Rad, Hercules, CA, USA) at 490 nm. All of the experiments were repeated at least three times.

### Colony forming assay

M7-TR and M7-FR cells were incubated for 24 h. Subsequently, the cells were treated with different concentrations of Huaier extract for 24 h. M7-TR and M7-FR cells in single-cell suspension were seeded in a 6-cm Petri dish (1500 cells per dish) of completed medium. The cells were cultured for another 14 days. The medium was refreshed every three days. Thereafter, the cells were washed with phosphate-buffered saline (PBS) and were fixed with paraformaldehyde and stained with 0.5% crystal violet. Only colonies containing >50 cells were counted. Images of clones were obtained using an Olympus digital camera (Olympus, Tokyo, Japan).

### Cell-cycle analysis

Cells at a density of 3 × 10^5^ cells/well were seeded into a 6-cm Petri dish and incubated with completed medium at 37 °C for 24 h. Subsequently, the cells were treated with Huaier extract for 48 h, and the total cells were collected. The cells were fixed with 75% cold ethanol (1 mL of PBS and 3 mL of absolute ethanol) at −20 °C overnight. Then, the DNA of cells was stained with 200 of μL RNase A (1 mg/mL) and 500 μL of propidium iodide (PI, 100 μg/mL) (Liankebio, Zhejiang, China) for 30 min at room temperature in the dark, and they were analysed using a FACScan flow cytometer. The data were analysed using ModFitLT software, version 2.0 (Becton Dickinson, Franklin Lakes, NJ, USA).

## Microrna and Transfection

MicroRNA mimics and the corresponding negative controls (NCs) were obtained from Guangzhou RiboBio (Guangzhou, China). To overexpress miR-203, we transfected mimics of miR-203 with Lipofectamine 2000 (Invitrogen) into M7-TR and M7-FR cells, according to the manufacturer’s protocol. Then, the cells were harvested after 48 h for mRNA and MTT and for transwell and protein analysis.

### Quantitative RT-PCR analysis

RNA was extracted using TRIzol (Takara, Dalian, China) reagents. Total RNA was used for RT reactions and quantitative (q)RT-PCR, according to the manufacturer’s protocol (Takara). The expression of U6 was used as an endogenous control for the analysis of miRNA expression, and GAPDH was used as an endogenous control for the analysis of other mRNA expression levels. The experiments were repeated in triplicate at a minimum.

### Western blot analysis

Cells were lysed with radio immunoprecipitation assay (RIPA) and PMSF (Biocolors, Shanghai, China) and were quantified using a BCA protein concentration assay kit (Merck, Darmstadt, Germany). Fifty micrograms of proteins were separated by 10% SDS-PAGE and were electro-blotted onto a PVDF membrane using a semidry blotting apparatus (Bio-Rad, Hercules, CA, USA). After blocking with 5% nonfat milk, the membranes were incubated with primary antibodies overnight at 4 °C. The next day, the membranes were labelled with secondary antibody, and signals were detected using a Luminescent Image analyser (GE Healthcare Bio-Sciences, Uppsala, Sweden). β-actin was used as the endogenous control.

### Xenograft tumourigenicity assay

Cells (5 × 10^6^ in 0.2 mL PBS) were injected subcutaneously into 4-week-old BALB/c nu/nu female mice (Taconic). After 2 days, the mice were randomly assigned to vehicle control (tri-distilled water) or Huaier extract alone (5 animals in each group). The Huaier group was administered 100 μL of solution containing 50 mg. Drugs were administered by gavage once every three days. Tumour growth was measured every 5 days, and tumour volume was calculated using the following equation: ${\rm{volume}}=({{\rm{width}}}^{2}\times {\rm{length}})/2$. After 40 days, the mice were sacrificed, and the xenografts were removed for immunohistochemical staining and western blot assays.

All of the animal care and experiments were conducted in accordance with the Guideline for Animal Experiments of Qilu Hospital and were approved by the Ethics Committee of Qilu Hospital for Animal Research (approval number KYLL-2016-221).

### Immunohistochemistry

The tumours in 4-week-old BALB/c nu/nu female mice previously injected with M7-TR and M7-FR cells were submitted to analysis. After excision, the tumour tissues were stored in 10% neutral-buffered formalin. After 24 h, the samples were paraffin-embedded and sliced into 4 μm sections. Immunohistochemistry was performed according to the manufacturer’s protocol. Tissue sections were then incubated with streptavidin–HRP complex, followed by haematoxylin. For negative controls, the antibody solution was replaced with PBS.

### Statistical analysis

The results were analysed using SPSS software (SPSS, Chicago, IL, USA). Student’s two-tailed t-test and one-way ANOVA were performed to determine significance. Each experiment was performed three times, and differences were considered significant when *p*-values < 0.05.

## References

[1] Chen W (2016). Cancer statistics in China, 2015. CA: a cancer journal for clinicians.

[2] Burstein HJ (2014). Adjuvant endocrine therapy for women with hormone receptor-positive breast cancer: american society of clinical oncology clinical practice guideline focused update. Journal of clinical oncology: official journal of the American Society of Clinical Oncology.

[3] Tamoxifen for early breast cancer: an overview of the randomised trials (1998). Early Breast Cancer Trialists’ Collaborative Group. Lancet.

[4] Poggio F (2016). Role of fulvestrant in the treatment of postmenopausal metastatic breast cancer patients. Expert review of clinical pharmacology.

[5] Fu X (2016). FOXA1 overexpression mediates endocrine resistance by altering the ER transcriptome and IL-8 expression in ER-positive breast cancer. Proceedings of the National Academy of Sciences of the United States of America.

[6] Zhang N, Kong X, Yan S, Yuan C, Yang Q (2010). Huaier aqueous extract inhibits proliferation of breast cancer cells by inducing apoptosis. Cancer science.

[7] Song X, Li Y, Zhang H, Yang Q (2015). The anticancer effect of Huaier (Review). Oncology reports.

[8] Wang X, Zhang N, Huo Q, Yang Q (2012). Anti-angiogenic and antitumor activities of Huaier aqueous extract. Oncology reports.

[9] Qi W (2016). Huaier extract synergizes with tamoxifen to induce autophagy and apoptosis in ER-positive breast cancer cells. Oncotarget.

[10] Ding X (2016). Radiosensitization effect of Huaier on breast cancer cells. Oncology reports.

[11] Li Y (2016). Huaier extract suppresses breast cancer via regulating tumor-associated macrophages. Scientific reports.

[12] Bean JR (2015). The PI3K/mTOR dual inhibitor P7170 demonstrates potent activity against endocrine-sensitive and endocrine-resistant ER+ breast cancer. Breast cancer research and treatment.

[13] Cook KL, Shajahan AN, Clarke R (2011). Autophagy and endocrine resistance in breast cancer. Expert review of anticancer therapy.

[14] Shah KN (2014). AKT-induced tamoxifen resistance is overturned by RRM2 inhibition. Molecular cancer research: MCR.

[15] Kim ST, Xu B, Kastan MB (2002). Involvement of the cohesin protein, Smc1, in Atm-dependent and independent responses to DNA damage. Genes & development.

[16] Halazonetis TD, Gorgoulis VG, Bartek J (2008). An oncogene-induced DNA damage model for cancer development. Science (New York, N.Y.).

[17] Bartkova J (2006). Oncogene-induced senescence is part of the tumorigenesis barrier imposed by DNA damage checkpoints. Nature.

[18] Zhou Y (2014). miR-203 induces oxaliplatin resistance in colorectal cancer cells by negatively regulating ATM kinase. Molecular oncology.

[19] Burma S, Chen BP, Murphy M, Kurimasa A, Chen DJ (2001). ATM phosphorylates histone H2AX in response to DNA double-strand breaks. The Journal of biological chemistry.

[20] Oakley GG (2001). UV-induced hyperphosphorylation of replication protein a depends on DNA replication and expression of ATM protein. Molecular biology of the cell.

[21] Lumachi F, Brunello A, Maruzzo M, Basso U, Basso SM (2013). Treatment of estrogen receptor-positive breast cancer. Current medicinal chemistry.

[22] Zhao M, Ramaswamy B (2014). Mechanisms and therapeutic advances in the management of endocrine-resistant breast cancer. World journal of clinical oncology.

[23] Cancer Genome Atlas N (2012). Comprehensive molecular portraits of human breast tumours. Nature.

[24] Musgrove EA, Sutherland RL (2009). Biological determinants of endocrine resistance in breast cancer. Nature reviews. Cancer.

[25] Thatte U, Bagadey S, Dahanukar S (2000). Modulation of programmed cell death by medicinal plants. Cellular and molecular biology.

[26] Kong X, Ding X, Yang Q (2015). Identification of multi-target effects of Huaier aqueous extract via microarray profiling in triple-negative breast cancer cells. International journal of oncology.

[27] Song YC, Xia W, Jiang JH, Wang QD (2005). [Reversal of multidrug resistance in drug-resistant cell line EAC/ADR by cepharanthine hydrochloride and its mechanism]. Yao xue xue bao = Acta pharmaceutica Sinica.

[28] Aogi K (1997). Overcoming CPT-11 resistance by using a biscoclaurine alkaloid, cepharanthine, to modulate plasma trans-membrane potential. International journal of cancer. Journal international du cancer.

[29] Harada T, Harada K, Ueyama Y (2012). The enhancement of tumor radioresponse by combined treatment with cepharanthine is accompanied by the inhibition of DNA damage repair and the induction of apoptosis in oral squamous cell carcinoma. International journal of oncology.

[30] Sun Y (2013). A polysaccharide from the fungi of Huaier exhibits anti-tumor potential and immunomodulatory effects. Carbohydrate polymers.

[31] Wang X (2013). Huaier aqueous extract suppresses human breast cancer cell proliferation through inhibition of estrogen receptor alpha signaling. International journal of oncology.

[32] Qi, W. *et al*. Huaier extract synergizes with tamoxifen to induce autophagy and apoptosis in ER-positive breast cancer cells. *Oncotarget* (2016). 10.18632/oncotarget.8303 PMC5041960 27027343

[33] Giacinti C, Giordano A (2006). RB and cell cycle progression. Oncogene.

[34] Pal, H. C. & Katiyar, S. K. Cryptolepine, a Plant Alkaloid, Inhibits the Growth of Non-Melanoma Skin Cancer Cells through Inhibition of Topoisomerase and Induction of DNA Damage. *Molecules* **21**, doi:10.3390/molecules21121758 (2016). 10.3390/molecules21121758 PMC6273109 28009843

[35] Yao J (2016). 53BP1 loss induces chemoresistance of colorectal cancer cells to 5-fluorouracil by inhibiting the ATM-CHK2-P53 pathway. Journal of cancer research and clinical oncology.

[36] Bueno RC (2014). ATM down-regulation is associated with poor prognosis in sporadic breast carcinomas. Annals of oncology: official journal of the European Society for Medical Oncology / ESMO.

[37] Rondeau S (2015). ATM has a major role in the double-strand break repair pathway dysregulation in sporadic breast carcinomas and is an independent prognostic marker at both mRNA and protein levels. British journal of cancer.

